# Deep Learning Reveals Liver MRI Features Associated With *PNPLA3* I148M in Steatotic Liver Disease

**DOI:** 10.1111/liv.70164

**Published:** 2025-06-06

**Authors:** Yazhou Chen, Benjamin P. M. Laevens, Teresa Lemainque, Gustav Anton Müller‐Franzes, Tobias Seibel, Carola Dlugosch, Jan Clusmann, Paul‐Henry Koop, Rongpeng Gong, Yuanyuan Liu, Niharika Jakhar, Feng Cao, Simon Schophaus, Thriveni Basavanapura Raju, Anastasia Artemis Raptis, Felix van Haag, Joel Joy, Rohit Loomba, Luca Valenti, Jakob Nikolas Kather, Titus J. Brinker, Moritz Herzog, Ivan G. Costa, Diego Hernando, Kai Markus Schneider, Daniel Truhn, Carolin V. Schneider

**Affiliations:** ^1^ Department of Medicine III University Hospital RWTH Aachen Aachen Germany; ^2^ Department of Diagnostic and Interventional Radiology University Hospital RWTH Aachen Aachen Germany; ^3^ Else Kroener Fresenius Center for Digital Health, Faculty of Medicine and University Hospital Carl Gustav Carus TUD Dresden University of Technology Dresden Germany; ^4^ Department of General Visceral and Transplantation Surgery University Hospital RWTH Aachen Aachen Germany; ^5^ MASLD Research Center, Division of Gastroenterology and Hepatology, Department of Medicine University of California San Diego California USA; ^6^ Department of Pathophysiology and Transplantation University of Milan Milan Italy; ^7^ Precision Medicine and Biological Resource Center Fondazione IRCCS Ca' Granda Ospedale Maggiore Policlinico Milan Italy; ^8^ Department of Medicine I, Faculty of Medicine and University Hospital Carl Gustav Carus TUD Dresden University of Technology Dresden Germany; ^9^ Medical Oncology, National Center for Tumor Diseases (NCT) University Hospital Heidelberg Heidelberg Germany; ^10^ Digital Biomarkers for Oncology German Cancer Research Center (DKFZ) Heidelberg Germany; ^11^ Institute for Computational Genomics RWTH Aachen Aachen Germany; ^12^ Department of Radiology University of Wisconsin Madison Wisconsin USA; ^13^ Department of Medical Physics University of Wisconsin Madison Wisconsin USA

**Keywords:** deep learning, medical imaging process, single nucleotide polymorphism, steatotic liver disease

## Abstract

**Background:**

Steatotic liver disease (SLD) is the most common liver disease worldwide, affecting 30% of the global population. It is strongly associated with the interplay of genetic and lifestyle‐related risk factors. The genetic variant accounting for the largest fraction of SLD heritability is *PNPLA3* I148M, which is carried by 23% of the western population and increases the risk of SLD two to three‐fold. However, identification of variant carriers is not part of routine clinical care and prevents patients from receiving personalised care.

**Methods:**

We analysed MRI images and common genetic variants in *PNPLA3, TM6SF2, MTARC1, HSD17B13* and *GCKR* from a cohort of 45 603 individuals from the UK Biobank. Proton density fat fraction (PDFF) maps were generated using a water‐fat separation toolbox, applied to the magnitude and phase MRI data. The liver region was segmented using a U‐Net model trained on 600 manually segmented ground truth images. The resulting liver masks and PDFF maps were subsequently used to calculate liver PDFF values. Individuals with (PDFF ≥ 5%) and without SLD (PDFF < 5%) were selected as the study cohort and used to train and test a Vision Transformer classification model with five‐fold cross validation. We aimed to differentiate individuals who are homozygous for the *PNPLA3* I148M variant from non‐carriers, as evaluated by the area under the receiver operating characteristic curve (AUROC). To ensure a clear genetic contrast, all heterozygous individuals were excluded. To interpret our model, we generated attention maps that highlight the regions that are most predictive of the outcomes.

**Results:**

Homozygosity for the *PNPLA3* I148M variant demonstrated the best predictive performance among five variants with AUROC of 0.68 (95% CI: 0.64–0.73) in SLD patients and 0.57 (95% CI: 0.52–0.61) in non‐SLD patients. The AUROCs for the other SNPs ranged from 0.54 to 0.57 in SLD patients and from 0.52 to 0.54 in non‐SLD patients. The predictive performance was generally higher in SLD patients compared to non‐SLD patients. Attention maps for *PNPLA3* I148M carriers showed that fat deposition in regions adjacent to the hepatic vessels, near the liver hilum, plays an important role in predicting the presence of the I148M variant.

**Conclusion:**

Our study marks novel progress in the non‐invasive detection of homozygosity for *PNPLA3* I148M through the application of deep learning models on MRI images. Our findings suggest that *PNPLA3* I148M might affect the liver fat distribution and could be used to predict the presence of *PNPLA3* variants in patients with fatty liver. The findings of this research have the potential to be integrated into standard clinical practice, particularly when combined with clinical and biochemical data from other modalities to increase accuracy, enabling easier identification of at‐risk individuals and facilitating the development of tailored interventions for *PNPLA3* I148M‐associated liver disease.

**Summary**

- Steatotic liver disease affects about 30% of people worldwide, and some people carry a gene variant called PNPLA3 I148M that increases the risk of developing this condition.
- In this study, we found that liver MRI scans combined with artificial intelligence can help predict whether a person carries this variant.
- This finding might help improve early diagnosis and stratified treatment in the future.

## 1 Introduction

Steatotic liver disease (SLD) represents a spectrum of liver disorders that includes metabolic dysfunction‐associated steatotic liver disease (MASLD), alcohol‐related liver disease (ALD), and metabolic dysfunction–alcohol‐associated liver disease (MetALD). Notably, MASLD is rapidly emerging as a major cause of liver‐related mortality and morbidity worldwide, affecting approximately 30% of the adult population [1]. In the United States, the prevalence of MASLD is forecast to increase by 21%, from 83.1 million cases in 2015 to 100.9 million by 2030 [2]. In addition, ALD is a major cause of chronic liver disease worldwide, accounting for 5.1% of all disease and injury globally [3, 4], while MetALD, which is characterised by the coexistence of metabolic dysfunction and significant alcohol consumption, represents a distinct clinical entity with its own implications for disease progression and treatment [5, 6]. Additionally, MASLD can progress to metabolic dysfunction‐associated steatohepatitis (MASH), potentially leading to cirrhosis and hepatocellular carcinoma (HCC) [7, 8, 9]. With this increasing prevalence of SLD, personalised prevention and treatment strategies have become crucial concerns.

Genetic factors play an important role in the development and progression of SLD, and genome‐wide association studies have shown that specific single nucleotide polymorphisms (SNPs) have a pivotal impact on SLD and its development [10]. For example, the presence of SNPs *HSD17B13* rs72613567_T and *MTARC1* rs2642438_A reduces the risk of developing conditions such as liver cirrhosis and HCC [10, 11, 12, 13]. On the other hand, the presence of SNPs *GCKR* rs1260326_T, *TM6SF2* rs58542926_T and *PNPLA3* I148M are associated with SLD progression [2, 12, 13]. Among them, the *PNPLA3* I148M variant accounts for the largest fraction of SLD heritability and is the most well‐studied genetic risk factor for SLD development and progression [14]. Individuals with SLD who possess the *PNPLA3* I148M variant, resulting in an isoleucine to methionine substitution at the amino acid position 148 (*PNPLA3* I148M), have a 220% higher likelihood of developing fibrosis and a 248.8% higher likelihood of developing MASH compared to those without the variant [14]. Personalised care, in the case of *PNPLA3* I148M, is especially interesting as the variant is common: 23%, 49% and 17% in patients of European, Hispanic and African American ancestry, respectively [12]. In a recent study of *PNPLA3* I148M homozygotes, a hepatocyte‐targeted N‐acetylgalactosamine (GalNac)–conjugated small interfering RNA was able to reduce liver fat after 1 month by 70% [15]. With these increasingly available therapeutic options, it is apparent that easy, readily available screening options are necessary.

Determining whether a patient carries these SNPs is essential for creating personalised prevention strategies that are specifically tailored to the individual's risk of disease progression [16]. However, the genetic testing for identification of SNPs, including *PNPLA3* I148M, has not yet been integrated into routine clinical practice due to the additional cost, extensive acquisition time and lack of the required infrastructure for routine genetic testing in some hospitals. Recent studies, such as Veldhuizen et al. [17], have demonstrated the feasibility of using deep learning models for cardiovascular event prediction from liver imaging. Additionally, Kather et al. [18] have demonstrated that deep learning models can capture imaging signatures associated with specific genetic variations on pathology slides [19]. Building on this concept, we hypothesised that the *PNPLA3* I148M variant may be detectable via subtle imaging signatures on liver MRI. This assumption is further supported by prior studies demonstrating that this variant is associated with distinct spatial patterns of hepatic fat accumulation [20, 21].

Given these challenges, our research proposes an innovative approach to bridge this gap, which can be applied when patients are already undergoing liver MRI examination for other clinical reasons. MRI is a highly accurate and practical technique for detecting liver abnormalities and assessing fat content [22, 23, 24]. Its advantages include being non‐invasive, free from radiation exposure, and highly precise in quantifying the proton density fat fraction (PDFF), a measurement of liver fat. These benefits make MRI a widely used tool in diagnosing and monitoring liver conditions. Incorporating the identification of carriers of high‐risk genetic variants into routine MRI could facilitate early intervention and more targeted treatments. The analysis of MRI imaging data can be facilitated through the application of big data techniques and deep learning models. The latter has developed very rapidly in recent times and has shown extraordinary potential [22], while transformer models, specifically, have been proven to be a compelling option for visual tasks [23]. We therefore hypothesised that genetic variants associated with hepatic steatosis might lead to zonal changes in hepatic steatosis that can be used to identify variant carriers using liver MRI.

## 2 Methods

### 2.1 Study Cohort and Design

This study is based on the UK Biobank dataset, which includes comprehensive genetic and health information from circa half a million UK participants. They were recruited between 2006 and 2010 from 22 assessment centres across Wales, Scotland, and England, providing a rich source of longitudinal data for research purposes [25]. In 2014, the UK Biobank project was expanded to include imaging for up to 100 000 participants, aiming to create the largest and most comprehensive collection of medical imaging data globally upon completion. This initiative involves rigorous and standardised multimodal scanning across Stockport, Newcastle, Reading, and Bristol in the UK. The study includes volunteers from the original UK Biobank cohort, aged 40 to 69 years [24]. The ethnicity of our study cohort was categorised into broad groups: White, Mixed, Asian, Black, and Other. While the UK Biobank classifies Chinese as a separate category, we included Chinese under the Asian category in this study. Participants who selected “Prefer not to answer” or “Do not know” for their ethnicity were not considered in the ethnicity count. In this study, we specifically focus on liver MRI data, utilising a multi‐echo spoiled gradient echo acquisition (Field ID 20254, *n* = 45 603).

Additionally, the UK Biobank also provides genotypic data for all participants. Variants were directly genotyped, not imputed. The dataset derived from two closely related genotyping arrays: The UK BiLEVE study involved 49 950 participants, genotyped at 807 411 markers, while the remaining 438 427 participants were genotyped on an array with 825 927 markers, sharing approximately 95% of the marker content with the UK BiLEVE array. Marker‐level quality control involved statistical tests across batches and arrays, leading to ~0.97% of genotype calls being set to missing. At the sample level, 968 samples (0.2%) with high missingness or abnormal heterozygosity and 652 individuals with sex discrepancies were flagged. The final dataset demonstrated high consistency (99.87% concordance in duplicates, *r* ^2^ = 0.93 with external allele frequencies) [26]. In this study, five SNPs that are associated with SLD progression were used as training and prediction labels, including *PNPLA3* I148M, *HSD17B13* rs72613567_T, *MTARC1* rs2642438_A, *GCKR* rs1260326_T and *TM6SF2* rs58542926_T.

**FIGURE 1:**
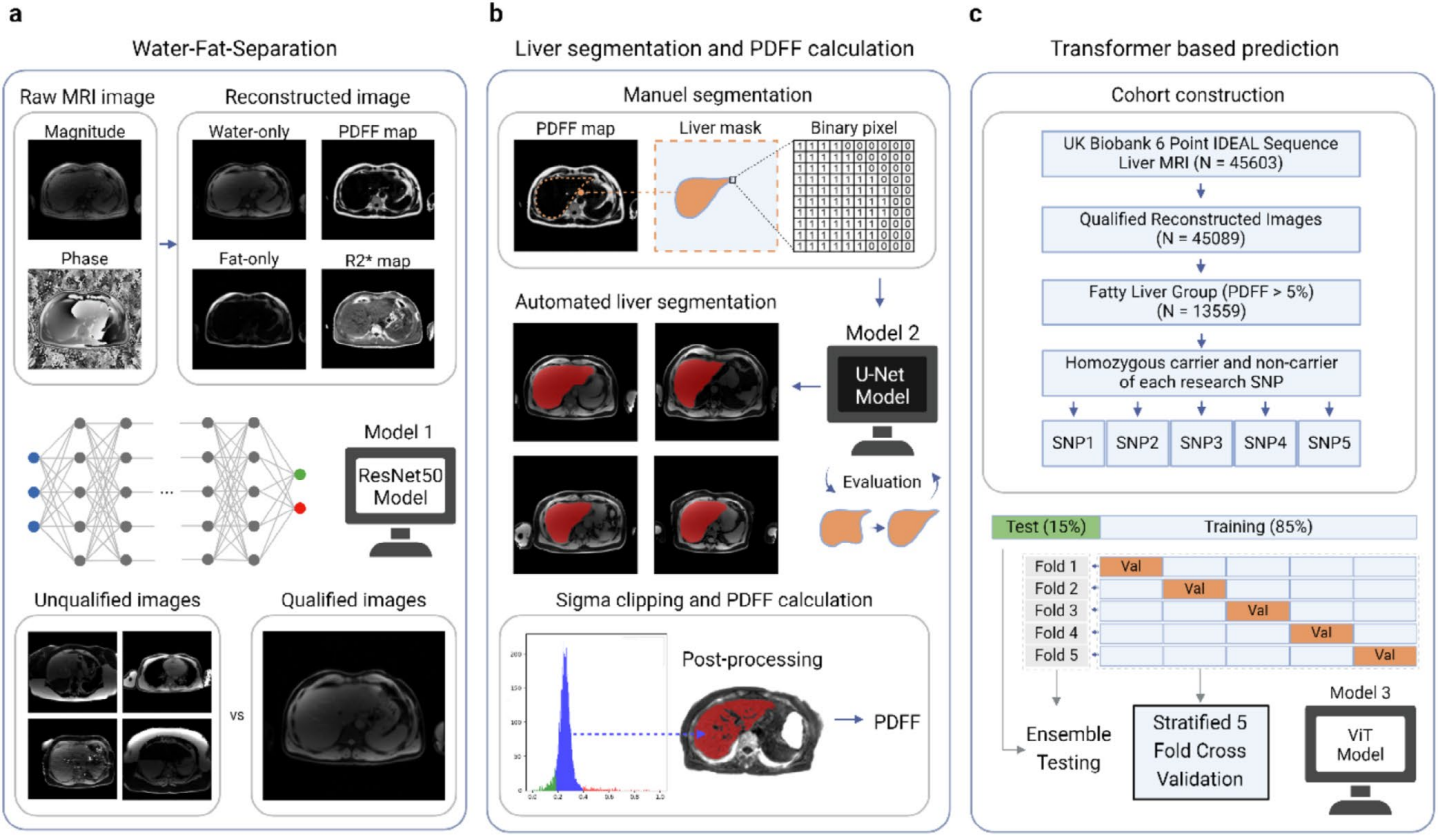
Deep learning‐based workflow for predicting steatosis‐associated SNPs using PDFF maps and liver segmentation on abdominal MRIs. (a) PDFF maps were obtained by utilising fat‐water imaging software. However, during the image reconstruction process, water‐fat swaps can occur, leading to incorrect mapping of water and fat signals. To address this, a ResNet [27] was trained to remove these artefacts and filter out unsuitable images. (b) To calculate the liver PDFF value, we trained a U‐Net to segment the liver region. We further refined the segmentations by sigma clipping to reduce the impact of blood vessels, cysts and other factors that could potentially affect the PDFF value. Finally, we calculated the median liver PDFF value. (c) Patients with median PDFF values greater than 5% constituted the study cohort. Homozygous carriers and non‐carriers of each SNP were selected for the corresponding study cohort. The final scores were obtained on the test set after using five‐fold stratified cross‐validation and ensemble testing method. For genetic variants with high test scores, subgroup analyses were performed based on obesity, sex and age. This figure was created in BioRender. Chen, Y. (2024) BioRender.com/w67o014. Reproduced by kind permission of UK Biobank.

**FIGURE 2:**
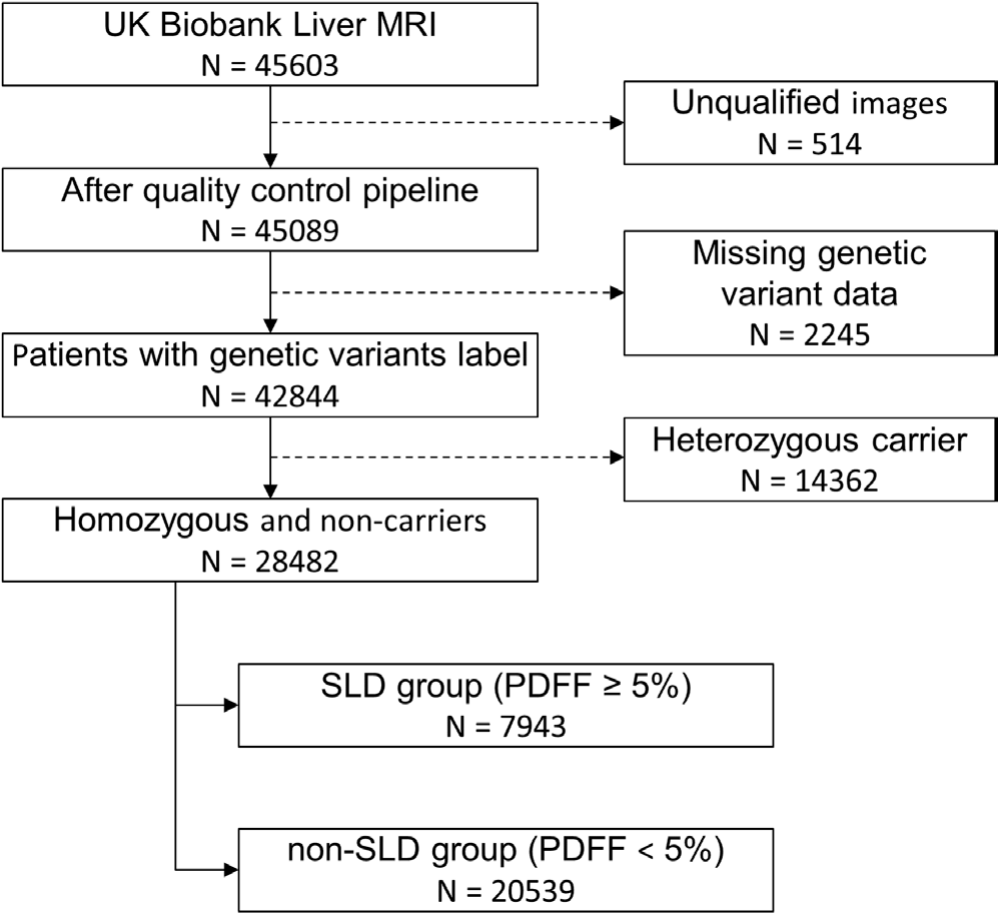
Exclusion criteria of our study cohort.

We proceeded as follows (Figure 1): firstly, we derived PDFF maps from the magnitude and phase MRI data (*n* = 45 603), based on a chemical shift‐based water‐fat separation method [28, 29, 30]. Unsuitable images (*n* = 514), including those with incorrect positioning, intensity abnormalities and water‐fat swaps, were identified and removed from the dataset. Subsequently, the liver region was extracted by a U‐Net segmentation model [31]. As we are focusing on the homozygosity of genetic variants, all heterozygous carriers (*n* = 14 362) and individuals without PNPLA3 I148M labeling (*n* = 2245) were excluded (Figure 2). Finally, our study cohort consisted of *n* = 28 482 individuals. Those with a median PDFF value exceeding 5% [32] were categorised as the SLD group (*n* = 7943), while patients exhibiting a PDFF value below 5% were categorised as the non‐SLD group (*n* = 20 539, Table 1). This stratification minimises potential biases, as PNPLA3 is already linked to an increased risk of SLD. By separately evaluating model performance in the SLD and non‐SLD groups, we aim to discern whether the model's predictive capacity reflects underlying genotype‐driven lipid distribution patterns rather than merely differentiating liver fat content. Finally, after benchmarking different machine learning algorithms (Figure S1), we selected a Vision Transformer image classification model to classify these patients based on segmented liver magnitude images and genetic variant labels. For each SNP, we selected non‐carriers and homozygous carriers from the study cohort to create the SNP‐based research cohorts (Table 2).

**TABLE 1:** Baseline characteristics of the study cohort.

Characteristic	SLD group	Non‐SLD group	*p*‐value
	*N* = 7943^a^	*N* = 20539^a^	
*Demographics*			
Ethnicity			0.034^b^
Asian	127 (1.6%)	253 (1.2%)	
Black	49 (0.6%)	170 (0.8%)	
Mixed	33 (0.4%)	95 (0.5%)	
Other	36 (0.5%)	113 (0.6%)	
White	7674 (97%)	19 855 (97%)	
Sex			< 0.001^b^
Female	3001 (38%)	11 713 (57%)	
Male	4942 (62%)	8826 (43%)	
Age (years)			0.062^b^
40–50	2213 (28%)	5718 (28%)	
51–60	3323 (42%)	8334 (41%)	
61–70	2388 (30%)	6451 (31%)	
BMI (kg/m2)	28.64 (26.27, 31.50)	25.09 (23.04, 27.50)	< 0.001^c^
*Clinical variables*			
PNPLA3 I148M			< 0.001^b^
Homozygous Carrier	961 (12%)	1059 (5.2%)	
Non‐Carrier	6982 (88%)	19 480 (95%)	
Obesity	2942 (37%)	2168 (11%)	< 0.001^b^
PDFF Median	0.08 (0.06, 0.12)	0.03 (0.02, 0.04)	< 0.001^c^
Type 2 Diabetes	960 (12%)	703 (3.4%)	< 0.001^b^
Mortality	109 (1.4%)	220 (1.1%)	0.033^b^
Alcohol Consumption (g/d)	8.31 (1.60, 17.83)	8.23 (1.95, 13.80)	< 0.001^c^
*Laboratory variables*			
ALT (U/L)	25.05 (18.76, 33.90)	18.17 (14.25, 23.60)	< 0.001^c^
AST (U/L)	25.60 (21.90, 30.60)	23.60 (20.40, 27.40)	< 0.001^c^
GGT (U/L)	33.05 (23.20, 49.40)	22.10 (16.40, 32.40)	< 0.001^c^
Cholesterol (mmol/L)	5.73 (4.99, 6.50)	5.66 (4.98, 6.38)	< 0.001^c^
Triglycerides (mmol/L)	1.86 (1.33, 2.63)	1.25 (0.92, 1.78)	< 0.001^c^

*Note:* In the SLD group, 37% of patients are obese, compared to only 11% in the non‐SLD group, indicating a significant difference. Additionally, the proportion of males in the SLD group is 62%, whereas it is only 43% in the non‐SLD group, also showing a significant difference. This trend is consistent in the ALT levels and alcohol consumption, both of which also show statistically significant differences.

^a^ *n* (%); Median (IQR).

^b^ P earson's Chi‐squared test.

^c^ W ilcoxon rank sum test.

**TABLE 2:** Characteristics of carriers of the different steatosis‐related SNPs groups.

Variable	PNPLA3 I148M			TM6SF2 (rs58542926_T)			GCKR (rs1260326_T)			MTARC1 (rs2642438_A)			HSD17B13 (rs72613567_T)		
	Homozygous carrier, *N* = 961^a^	Non‐carrier, *N* = 6,982^a^	*p*‐value^b^	Homozygous carrier, *N* = 137^a^	Non‐carrier, *N* = 10465^a^	*p*‐value^b^	Homozygous carrier, *N* = 2191^a^	Non‐carrier, *N* = 4481^a^	*p*‐value^b^	Homozygous carrier, *N* = 989^a^	Non‐carrier, *N* = 6574^a^	*p*‐value^b^	Homozygous carrier, *N* = 1041^a^	Non‐carrier, *N* = 6880^a^	*p*‐value^b^
PDFF Median	0.095 (0.068, 0.154)	0.078 (0.060, 0.119)	< 0.001	0.123 (0.074, 0.186)	0.079 (0.061, 0.120)	< 0.001	0.084 (0.062, 0.130)	0.080 (0.061, 0.123)	0.006	0.079 (0.061, 0.117)	0.082 (0.062, 0.128)	0.023	0.085 (0.062, 0.130)	0.081 (0.061, 0.124)	0.041
Age (years) *n* (%)			0.228			0.22			0.196			0.955			0.674
40–50	250 (26%)	1963 (28%)		39 (29%)	2901 (28%)		624 (29%)	1266 (28%)		269 (27%)	1780 (27%)		280 (27%)	1902 (28%)	
51–60	399 (42%)	2924 (42%)		64 (47%)	4318 (41%)		865 (40%)	1860 (42%)		409 (41%)	2753 (42%)		443 (43%)	2835 (41%)	
61–70	309 (32%)	2079 (30%)		33 (24%)	3229 (31%)		699 (32%)	1344 (30%)		308 (31%)	2025 (31%)		314 (30%)	2134 (31%)	
BMI (kg/m^b^), Median (IQR)			< 0.001			0.002			< 0.001			0.357			0.488
Not obese (< 30)	672 (70%)	4324 (62%)		105 (77%)	6655 (64%)		1478 (67%)	2812 (63%)		627 (63%)	4262 (65%)		677 (65%)	4396 (64%)	
Obese (≥ 30)	289 (30%)	2653 (38%)		32 (23%)	3799 (36%)		712 (33%)	1664 (37%)		362 (37%)	2305 (35%)		363 (35%)	2474 (36%)	
Sex, *n* (%)			0.438			0.044			0.383			0.094			0.431
Female	374 (39%)	2627 (38%)		64 (47%)	4006 (38%)		828 (38%)	1743 (39%)		357 (36%)	2556 (39%)		388 (37%)	2652 (39%)	
Male	587 (61%)	4355 (62%)		73 (53%)	6459 (62%)		1363 (62%)	2738 (61%)		632 (64%)	4018 (61%)		653 (63%)	4228 (61%)	
ALT (U/L), Median (IQR)	26.440 (19.160, 38.360)	24.920 (18.730, 33.370)	< 0.001	24.980 (18.920, 35.720)	25.020 (18.660, 34.245)	0.81	24.665 (18.540, 35.015)	24.860 (18.560, 34.365)	0.84	25.220 (18.730, 33.850)	25.360 (18.950, 35.220)	0.376	24.080 (18.495, 33.470)	25.595 (18.800, 35.450)	0.008
Alcohol consumption (g/d), Median (IQR)	8.229 (1.714, 16.914)	8.314 (1.557, 17.829)	0.58	8.314 (1.367, 19.200)	8.314 (1.514, 17.829)	0.9852	8.229 (1.343, 16.629)	8.400 (1.960, 17.979)	0.0052	8.657 (1.371, 18.000)	8.229 (1.457, 16.893)	0.23	9.600 (2.743, 18.214)	8.229 (1.323, 16.736)	< 0.001
Mortality status *n* (%)	10 (1.0%)	99 (1.4%)	0.346	2 (1.5%)	128 (1.2%)	0.6864	23 (1.0%)	52 (1.2%)	0.6873	16 (1.6%)	82 (1.2%)	0.337	14 (1.3%)	84 (1.2%)	0.736

*Note:* We analysed the homozygous carriers and non‐carriers for various SNPs. The *PNPLA3* I148M variant showed a highly significant difference in PDFF values, BMI, and ALT levels (*p* < 0.001), whereas the other SNP groups exhibited differences in individual variables. This demonstrates why *PNPLA3* I148M has the best predictive performance in our results, and also highlights that PDFF values, BMI, and ALT are critical variables for distinguishing between homozygous carriers and non‐carriers of *PNPLA3* I148M.

^a^ Median (IQR); *n* (%).

^b^ *p*‐value, Continuous variables: Wilcoxon rank‐sum test. Categorical variables: Pearson's Chi‐squared test.

### 2.2 MRI acquisition and Post‐Processing

Images were acquired at the Biobank Imaging Centre at Cheadle (UK) using a Siemens 1.5 T MAGNETOM Aera scanner. For each patient, six dynamic sequence acquisitions were performed, following a dynamic imaging protocol in which six sequential image series were acquired, spaced 1.6225 s apart. Each series includes images acquired at six different echo times. For each echo time, a magnitude image and its corresponding phase image were captured as single transverse slices during end‐expiration breath‐hold, without the use of a contrast agent injection. The slices were positioned at the porta hepatis, with the following scan parameters: TR = 14 ms, TE = 1.2/3.2/5.2/7.2/9.2/11.2 ms, FA = 5°, bandwidth = 1565 Hz, voxel size 1.719 × 1.719 × 10.0 mm, 256 × 232 matrix.

For the present study, we utilised the first of six acquired dynamic sequences, comprising six magnitude images and six phase images, differentiated by varying TE values. PDFF maps were generated using the aforementioned MATLAB‐based fat‐water imaging software, which employs a previously validated confounder‐corrected mapping method, including correction for R2* effects and a multi‐peak fat spectral model [28, 29, 30].

However, some images were not adequately suitable for the task due to issues with the original dataset's quality and the water‐fat separation reconstruction process. In this study, these unqualified images were defined as follows: (i) As per the UK Biobank imaging enhancement protocol, the slices should be positioned at the porta hepatis [24]. However, some slices were positioned too high or too low, leading to the absence of the liver or the presence of only a small part of the liver in the image; (ii) Intensity abnormalities: The image intensity was either too high or too low, making it difficult to identify the liver region; (iii) Another problem is the water‐fat swap. This artefact occurs during the water‐fat separation reconstruction process, where the signals for water and fat are incorrectly swapped. As a result, regions that should appear as fat may appear as water, and vice versa. This misalignment can lead to substantial errors in image interpretation, particularly in the accurate assessment of tissue composition.

To mitigate the issues associated with these unqualified images, we employed an automated filtering process using a ResNet50 image classification model. To validate the model's accuracy, we recorded the predictions, examining the probability that each image is classified as a suitable image. Images, predicted with probabilities below 0.8 were manually reviewed. Furthermore, we conducted an additional quality control step by manually evaluating 100 randomly selected samples from each of the probability ranges [0.8, 0.9] and [0.9, 1.0], to monitor the model's performance. Unsuited images identified through this process, along with an equal number of qualified images, were added to the training set as negative and positive samples, respectively. The model was then retrained to enhance its learning efficacy. This refinement continued iteratively until no further unqualified images were detected. We eventually used 7 iterations, with the results evaluated by a medical imaging scientist (Y.C., 2 years of experience) and reviewed by a senior radiologist (D.T., 10+ years of experience). Finally, we compiled a dataset consisting of 45 089 qualified images, discarding 514 unqualified images.

### 2.3 Liver Segmentation and PDFF Value Calculation

Liver segmentations were obtained using a U‐Net‐based segmentation model. The training data was manually segmented by a medical imaging scientist (Y.C., 2 years of experience) and reviewed by a senior radiologist (D.T., over 10 years of experience).

The model was initially trained using 300 manually segmented images and then tested on 100 randomly selected samples. The predicted segmentations of these samples were visually evaluated, and any inaccuracies were manually corrected. These corrected segmentations were subsequently added to the training dataset for the next iteration of training. This iterative model refinement process continued until the predicted segmented liver segmentations aligned with the liver contours, as verified through visual inspection. A total of 309 corrected segmentations were added to the training dataset. The final model was trained with 609 manually segmented images, while the validation set comprised 68 manually segmented images.

Upon successful training and validation, the model was employed to perform liver segmentation across the entire study cohort. Subsequently, these liver segmentation images were overlaid on the corresponding PDFF maps to derive liver PDFF maps. To enhance the reliability of the liver PDFF values, we employed sigma clipping (iteration = 5) on the segmented regions. We excluded air cavities (upper threshold, sigma = 2), blood vessels, and cysts (lower threshold, sigma = 2) based on the R2* map and outlier values in the PDFF map (upper threshold, sigma = 1.1), as these structures could potentially skew the PDFF readings. The median pixel value of the post‐processed liver PDFF maps was then calculated to establish the liver PDFF value for each patient.

### 2.4 Predictive Model Training and evaluation

For this study, we selected the sixth image in the sequence (TE = 11.2 ms) to train our deep learning classification model. The 11.2 ms echo time places the image in an out‐of‐phase condition, which provides the clearest distinction between SLD and non‐SLD, by partial cancellation of water and fat signals respectively, thereby enhancing the visibility of fat within the liver [33]. This increased contrast is particularly beneficial for accurately identifying and assessing the extent of steatosis, making it an optimal choice for this study. To ensure compatibility with the Vision Transformer model, a bounding box that minimally encloses the liver region was drawn, based on the liver segmentation. Lastly, extra padding was added, transforming the rectangle into a square bounding box. The region within the bounding box was extracted and subsequently resized to 224 × 224 pixels, employing cubic interpolation. The cubic interpolation is preferred in this case as it provides smoother and more accurate results compared to linear interpolation, especially when scaling small images to larger sizes by considering the surrounding pixel values in a 4 × 4 neighbourhood.

The images were processed using the Vision Transformer (ViT‐base‐patch16‐224) model, available from the Hugging Face model repository [34]. The model was originally pre‐trained on ImageNet‐21 k, a dataset containing 14 million images across 21 000 classes, and later fine‐tuned on ImageNet, which comprises 1 million images and 1000 classes [35]. The outcome variable for our analysis was defined in a binary manner: individuals were classified as positive if they were homozygous for the PNPLA3 I148M variant, and as negative if they were non‐carriers. All heterozygous individuals were removed from the dataset to establish a clear distinction between groups (Figure 2). Due to the imbalance between positive and negative samples, all cohort data were partitioned according to the following protocol: we employed a stratified method to randomly extract 15% of the samples as an internal test set, ensuring consistent label distribution across the subsets. The internal test set was excluded from the training process. To maximise the utilisation of the dataset and reduce bias from random allocation, the remaining 85% of the data underwent a five‐fold stratified cross‐validation. The optimal model from each fold was subsequently used to evaluate the internal test set through an ensemble testing method. All reported results in this study reflect the outcomes on the test sets, and the 95% confidence intervals were calculated using the bootstrap method.

To visualise the model's focus areas, attention maps were generated during the test phase by extracting weights from the final attention head of the last layer of the Vision Transformer model. These maps were resized to match the input image dimensions and overlaid on the original images to highlight the regions of interest. Further, we identified common samples across all folds and averaged their attention maps to obtain a consistent representation. The averaged attention maps were used to mitigate the variability introduced by the different folds and provide a clearer understanding of the model's attention mechanism.

### 2.5 Ethical Considerations

The study was performed in accordance with the Declaration of Helsinki. All participants provided informed consent, and the UKB project received ethical approval from the National Health Service National Research Ethics Service (Ref 11/NW/0382).

### 2.6 Statistical Analysis

Our main analyses were conducted using Python 3.10.13 (https://www.python.org/) for programming, and R version 4.3.2 (https://www.r‐project.org/) for result visualisation. For the Matlab‐based fat‐water imaging software we used Matlab R2023b (The MathWorks, Natick, MA). To assess model performance, we used the area under the receiver operating characteristic curve (AUROC) as our evaluation metric. Confidence intervals were calculated using the bootstrap method, with 95% confidence intervals. Predicted positive and negative samples were determined using a dynamic threshold by maximising Youden index. For all tables, *p*‐values were calculated using Wilcoxon rank sum test for continuous variables and Pearson's chi‐squared test for discrete variables. The significance level for all statistical tests was set at 0.05.

## 3 Results

### 3.1 Vision Transformer Models predict PNPLA3 I148M among well‐known Steatosis‐Associated SNPs on Liver MRIs in SLD

We hypothesised that genetic variants associated with hepatic steatosis might lead to zonal changes in hepatic steatosis, which can be used to identify variant carriers using liver MRIs. Therefore, we evaluated the performance of our Vision Transformer model in predicting the homozygosity of five SNPs in all patients, patients with SLD and patients without SLD (Table 1). The model's performance was quantified using the AUROC for each SNP (Figure 1). Notably, *PNPLA3* I148M exhibited the highest predictive accuracy in the SLD group with an AUROC of 0.68 (0.64–0.73). This trend was also consistent in the all‐patients group and non‐SLD group, with homozygosity for *PNPLA3* I148M still exhibiting the highest predictive accuracy with an AUROC of 0.61 (0.57–0.64) and 0.57 (0.52–0.61), respectively. The results for all variants indicated that the prediction performance is generally higher in the SLD group compared to the non‐SLD group (Figure S2). Only homozygosity for the *PNPLA3* I148M variant in the SLD group achieved an AUROC of 0.68, indicating that the model was able to learn some relevant information associated with this variant in the SLD population.

**FIGURE 3:**
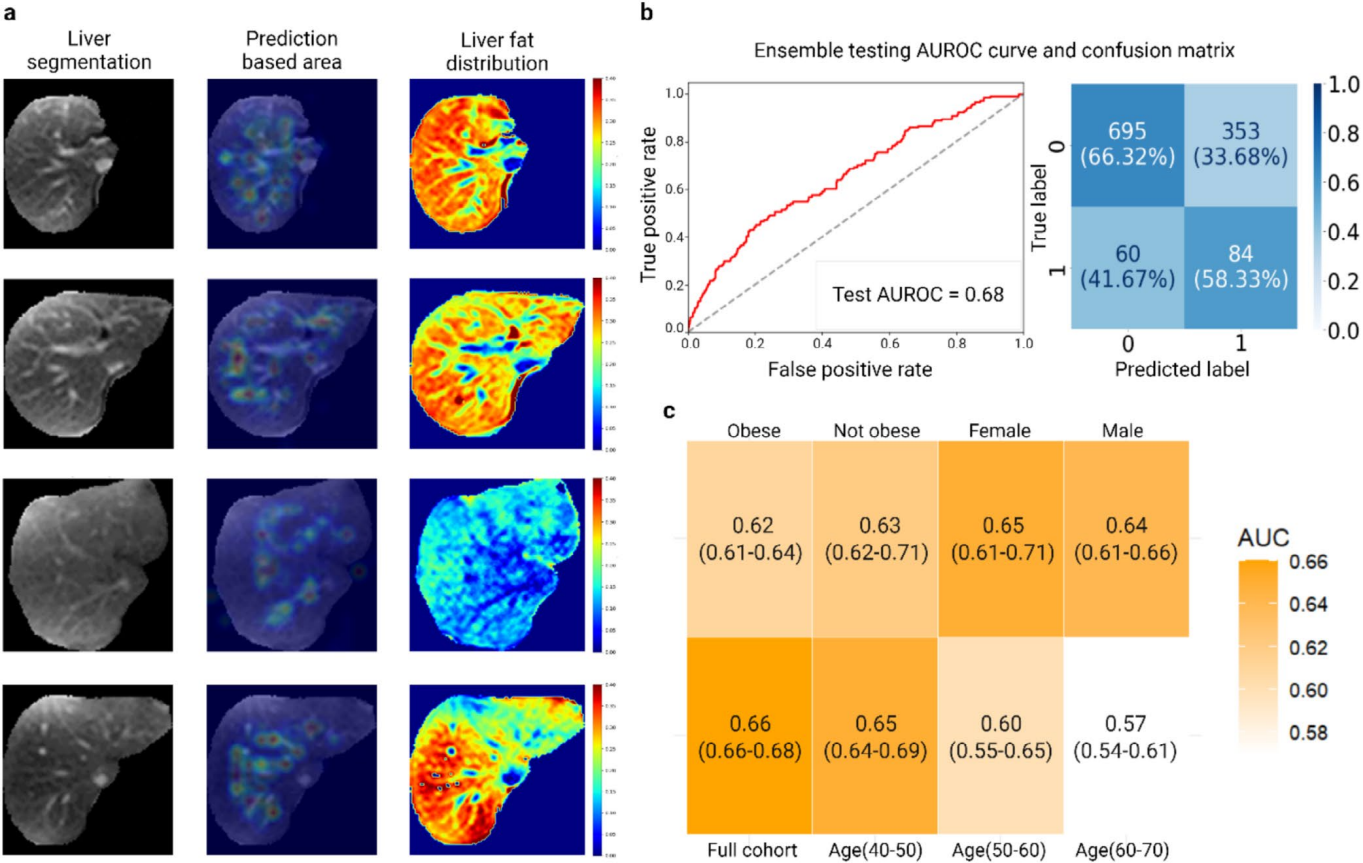
Visualisation of prediction‐contributing areas of *PNPLA3* I148M carriers on liver MRIs in SLD group. (a) We selected true positive samples from the predictions of *PNPLA3* I148M model by inference on the test set. Each sample includes the liver segmentation based on the magnitude image, an attention map highlighting the regions contributing to the prediction, and the distribution of hepatic fat in these areas. (b) AUROC and confusion matrix of *PNPLA3* I148M model using the ensemble testing method. (c) The heatmap of AUROCs, resulting from the subgroup analysis based on obesity (BMI > 30), sex and age. This plot shows one of six attempts using different random seeds for data splitting. For each subgroup, we presented the AUROC from the current attempt, along with the minimum and maximum values (min‐max) observed across all attempts. This figure was created in BioRender. Chen, Y. (2024) BioRender.com/g20c852. Reproduced by kind permission of UK Biobank.

Additionally, the *PNPLA3* I148M variant demonstrated a sensitivity of 0.72 and a specificity of 0.58 within the SLD group, while maintaining the same sensitivity of 0.72 but showing a markedly lower specificity of 0.43 in the non‐SLD group. These findings indicate that, although the homozygosity for the variant was equally detected in both groups, the ability to correctly identify non‐carriers is reduced in the non‐SLD population. This disparity in specificity may suggest that the PDFF value influences the accuracy of variant detection. To investigate the potential influence of confounding factors, we further divided the SLD group into subgroups based on BMI, sex and age (Figure 3c). In younger patients and in females, the AUROC for predicting *PNPLA3* I148M was highest [36] (Figure 3c).

### 3.2 Model Predicts PNPLA3 I148M Variants Instead of Traditional Risk Factors of Obesity and Type 2 Diabetes

To test whether our model was merely detecting general risk factors, we used our original model, which was trained by PNPLA3 I148M label, to predict various risk factor labels, such as obesity (BMI ≥ 30) and Type 2 diabetes. For obesity, the model yielded an AUROC of 0.35, and for Type 2 diabetes, an AUROC of 0.47—both values substantially lower than the AUROC of 0.68 observed for PNPLA3 I148M homozygosity. Considering the potential combined influence of these risk factors on the model's performance, we created two additional composite labels. The first composite label was defined as positive if either the diabetes label or the obesity label was positive, while the second composite label was defined as positive only when both obesity and diabetes were present. When predicting these new labels, the model achieved AUROCs of 0.37 and 0.39, respectively (Table S1). These results consistently indicate that our model is capturing genotype‐specific imaging signatures rather than relying on general indicators of hepatic steatosis or risk factors like obesity and Type 2 diabetes.

### 3.3 Higher PDFF and ALT Levels are Associated With Successful PNPLA3 I148M Variant Detection on Liver MRIs

Subsequently, we focussed on *PNPLA3* I148M and conducted an analysis of the common True Positive (TP) and False Negative (FN) samples of all folds predicted by the *PNPLA3* I148M model during cross‐validation to understand which patients' characteristics lead to better predictability. Our findings indicate that the median PDFF value for TP samples was 11% versus 8% in the FN samples (*p* = 0.004), and the median ALT value is 28 vs. 22 U/L in the FN samples (*p* = 0.045) (Table 3). For TP samples, specifically homozygous carriers of the *PNPLA3* I148M variant, higher PDFF values are associated with a greater likelihood of the model correctly classifying them as positive. Additionally, there was a notable difference in BMI between TP and FN samples, with 84% of the TP samples being non‐obese individuals, compared to 65% in the FN samples (*p* = 0.039, Table 3). These findings suggest that higher PDFF and ALT values are important indicators for identifying individuals with the *PNPLA3* I148M variant, thus strengthening our hypothesis that the prediction is based on zonal changes in hepatic fat (Table 3).

**TABLE 3:** Comparison of true positive and false negative predicted *PNPLA3* I148M carriers.

Variable	False negative, *N* = 31^a^	True positive, *N* = 51^a^	*p*‐value^b^
PDFF Median	0.076 (0.061, 0.098)	0.113 (0.069, 0.201)	0.004
Age (years), *n* (%)			0.37
40–50	7 (23%)	10 (20%)	
51–60	14 (45%)	30 (60%)	
61–70	10 (32%)	10 (20%)	
BMI (kg/m^2^), Median (IQR)			0.039
Not obese (< 30)	20 (65%)	43 (84%)	
Obese (≥ 30)	11 (35%)	8 (16%)	
Sex, *n* (%)			0.55
Female	13 (42%)	18 (35%)	
Male	18 (58%)	33 (65%)	
ALT (U/L), Median (IQR)	21.615 (15.910, 30.525)	27.570 (22.340, 36.533)	0.045
Alcohol consumption (g/d), Median (IQR)	7.314 (2.900, 11.043)	6.857 (2.271, 14.100)	> 0.99
Died during follow up, *n* (%)			
No	31 (100%)	51 (100%)	

^a^ Median (IQR); *n* (%).

^b^ *p*‐value, Continuous variables: Wilcoxon rank‐sum test. Categorical variables: Pearson's Chi‐squared test.

### 3.4 Sensitivity Analysis Using Decomposed Signals and Alcohol Consumption Status

**FIGURE 4:**
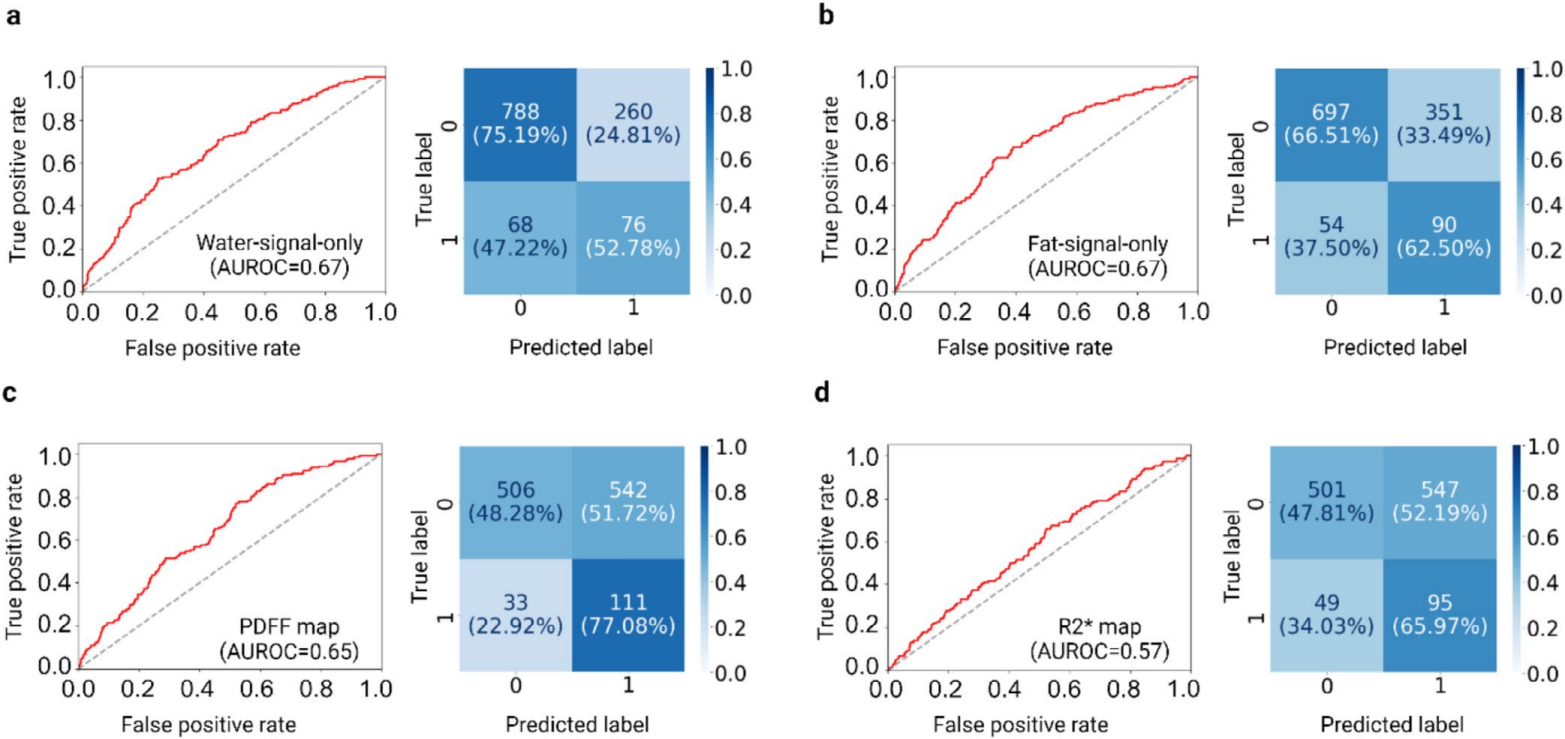
Comparative analysis of image modalities for detecting *PNPLA3* I148M homozygosity on liver MRIs. To investigate the specific patterns that distinguish *PNPLA3* I148M homozygosity in the liver region, we extended our analysis beyond the original magnitude images to include the water‐signal‐only image, fat‐signal‐only image, PDFF map, and R2* map. (a) The model using water‐signal‐only image achieved an AUROC of 0.67 (0.63–0.72), exhibiting the ability to detect negative samples but showing less effectiveness in identifying positive samples. (b) The model, using a fat‐signal‐only image, demonstrated an AUROC of 0.67 (0.63–0.73). Compared to the water‐signal‐only image, this model showed improved performance in detecting positive samples, although its ability to identify negative samples slightly decreased. (c) The model, using the PDFF maps, reached an AUROC of 0.65 (0.61–0.70). This model further enhanced the detection of positive samples. However, it frequently predicted false positives, likely due to a bias towards predicting positive samples. (d) The model using R2* map, which reflects the rate of signal decay influenced by local magnetic field inhomogeneities and often related to iron content in the liver, achieved an AUROC of 0.57 (0.52–0.62). This model produced a high number of false positives and exhibited a relatively low AUROC, suggesting that the R2* map patterns are not valuable for this specific task. Overall, both the fat‐signal‐only and water‐signal‐only images contain patterns that can distinguish *PNPLA3* I148M homozygous carriers from non‐carriers. The performance of these models is comparable to that of the magnitude images. This figure was created in BioRender. Chen, Y. (2024) BioRender.com/j91q961.

To analyse whether the prediction in these specific areas is based on steatosis as opposed to fibrosis, we conducted a sensitivity analysis. Here, we split the images into the water only signals, fat only signals, the PDFF map and the R2* map (can be used as a marker of fibrosis), which yielded AUROCs of 0.67 (0.63–0.72), 0.67 (0.63–0.73), 0.65 (0.61–0.70), 0.57 (0.52–0.62), respectively. These results indicate that the water and fat signals contribute most strongly to predicting *PNPLA3* I148M status, whereas the R2* map contributes minimally (Figure 4).

We further performed a sensitivity analysis excluding individuals with excessive alcohol consumption, defined as > 60 g/day for male and > 50 g/day for female, based on the 2024 EASL‐EASD‐EASO Clinical Guidelines on the management of MASLD [37]. Using the UK Biobank 24‐h alcohol intake data, we identified *n* = 100 male and *n* = 21 female in the SLD group who exceeded this threshold, and *n* = 431 individuals with missing values (Table S2). After excluding these individuals and retraining our model, the AUROC in the SLD group remained stable at 0.67 (0.63–0.70). The result showed a slight decrease but remained stable, indicating that the model's ability to distinguish PNPLA3 I148M homozygous carriers from non‐carriers is not primarily driven by alcohol‐related hepatic fat accumulation.

### 3.5 Predictive Patterns of PNPLA3 I148M on Liver MRIs

To finally understand if our predictions were based on steatosis, we further visualised attention maps of those TP samples to identify the key regions contributing to the model's predictions (Figure 3a). Interestingly, when comparing the attention maps with the liver fat distribution images, we found that regions with high fat content were not always the most influential contributors to the prediction results. Some areas with relatively high fat content still exhibited no predictive value. Instead, the attention maps highlighted regions with high liver fat content, which were mainly in the central part of the liver and particularly in areas adjacent to blood vessels, especially around the terminal vessels (Figure 3a). This suggests that the model's predictive power is influenced by the specific location of liver fat. It is possible that these regions provide more information to distinguish between *PNPLA3* I148M variant homozygous carriers and non‐carriers compared to areas with generally high PDFF values.

## 4 Discussion

In this study, we demonstrate that a deep learning model applied to liver MRI images distinguishes *PNPLA3* I148M homozygous carriers from non‐carriers in a SLD cohort. In individuals with SLD, defined as a PDFF of 5% or higher, our model achieved an AUROC of 0.68 (0.64–0.73), whereas the performance in the full‐cohort group and non‐SLD group was lower at an AUROC of 0.61 (0.57–0.64) and 0.57 (0.52–0.61), respectively. Among the five steatosis‐associated genetic variants evaluated, PNPLA3 I148M consistently exhibited the highest discriminative ability. These results suggest that the PNPLA3 I148M variant is associated with specific imaging signatures that reflect genotype‐related patterns of hepatic fat distribution.

Since AUROC is a metric that measures the ability of a model to distinguish between positive and negative classes, it summarizes the trade‐off between true positive and false positive rates across different threshold settings. An AUROC of 0.5 indicates that a model's ability to distinguish between positive and negative samples is no better than random guessing. When a model's AUROC falls below 0.6, it suggests that the model has learned little to no useful information from the training data. In the SLD cohort, our deep learning model distinguished PNPLA3 I148M homozygous carriers from non‐carriers with an AUROC of 0.68 (0.64–0.73), compared to an AUROC of 0.57 (0.52–0.61) in the non‐SLD cohort. This difference indicates that the imaging signatures associated with PNPLA3 I148M are likely reflecting a unique pattern of hepatic fat distribution, which are more prominent in individuals with steatosis. Furthermore, when comparing across five steatosis‐associated genetic variants, only PNPLA3 I148M yielded a notably higher AUROC. These observations suggest that the model is capturing specific genotype‐associated imaging patterns rather than merely detecting general risk factors for hepatic steatosis, thereby reinforcing the established association between PNPLA3 I148M and increased liver fat accumulation. Although it may still be suboptimal for allowing direct implementation in clinical practice without confirmation by genetic testing, the relatively narrow confidence interval of 0.64–0.73, however, suggests that the estimate is reasonably precise, meaning the true AUROC is likely to fall within this range, but there is still some variability.

The findings of our study highlight the moderate potential of Vision Transformer models to predict the presence of the PNPLA3 I148M variant on liver MRI, achieving an AUROC of 0.68. The superior predictive accuracy observed for PNPLA3 I148M in the SLD group highlights the variant's pivotal role in liver fat accumulation, which aligns with existing literature, emphasising the influence of PNPLA3 on liver fat accumulation and consequently on fibrosis [12, 14]. This association suggests that routine liver MRI of SLD patients could be used to identify a population that might benefit from *PNPLA3* I148M screening, hence identifying individuals at higher risk of developing more severe liver disease phenotypes. This is particularly useful as carriers of *PNPLA3* I148M are common (23% in Europeans, 49% in Hispanics and 17% African‐americans [12]) and targeted therapies for people with fibrotic MASH homozygous for this variant are under evaluation in early phase clinical studies [15]. If untreated, *PNPLA3* I148M homozygosity can lead to a three to four‐fold increased risk of MASH, cirrhosis and up to a 12 times elevated risk of HCC [38]. Several studies have robustly associated this variant with an increased risk of fat accumulation, liver inflammation and fibrosis [12, 39]. Additionally, the homozygosity for this variant profoundly impacts liver fibrosis severity, highlighting its role in disease progression [40].

Our analysis of the TP and FN samples provides further insights into the factors influencing predictive success. The significantly higher PDFF and ALT values in TP samples compared to FN samples indicate that higher hepatic fat levels lead to better predictions. This finding is consistent with previous studies that have identified elevated liver fat content and liver enzyme levels as being linked to PNPLA3 as indicative of hepatic steatosis and fibrosis [12, 41]. Furthermore, the difference in BMI distribution between TP and FN samples suggests that non‐obese individuals with the *PNPLA3* I148M variant may exhibit distinct metabolic or imaging characteristics that facilitate accurate identification (Table 3). This is particularly interesting as lean MASLD remains insufficiently understood [42]. Therefore, integration of clinical features, including for example, BMI and aminotransferases into MRI‐based assessment may further improve the ability to identify people homozygous for the PNPLA3 variants, who may benefit from targeted clinical management.

We observed imaging patterns in liver MRIs that may help differentiate *PNPLA3* I148M homozygous carriers from non‐carriers with moderate discriminative performance. These patterns are related to regions of elevated liver fat, adjacent to blood vessels, especially around the terminal vessels. An explanation might be derived from the different functionalities of the vessels. The vessels in the liver hilum primarily consist of the portal vein, which brings all digested foods and metabolites from the gut to the liver, whereas the capillary network of the terminal vessels is densely distributed within the liver tissue, enabling the transfer of oxygen, nutrients, and metabolic products from the blood to the hepatocytes. Here, an interaction with *PNPLA3* I148M might lead to more fat deposition in the areas surrounding the terminal vessels.

PNPLA3 encodes the patatin‐like phospholipase domain‐containing protein 3, which plays a crucial role in lipid remodelling and affects triglyceride hydrolysis [12]. The I148M variant results in decreased enzymatic activity and determines the ability to inhibit ATGL/PNPLA2, therefore impairing lipid metabolism and leading to increased triglyceride accumulation in hepatocytes [14, 43]. While the effects of the *PNPLA3* I148M variant occur in all hepatocytes, regions adjacent to terminal vessels, which have a rich blood supply and high metabolic activity, may show distinct patterns. These regions are exposed to higher insulin concentrations, as PNPLA3 expression is induced by insulin and cleared via insulin receptor (INSR) in hepatocytes [44]. Therefore, we hypothesise that the intracellular triglyceride storage, caused by the *PNPLA3* I148M variant, may form visible, specific patterns in these metabolically active regions that can be captured by MRI and detected by our model. This hypothesis needs to be validated in further studies.

One of our study's limitations is that the reached conclusions may not be applicable to non‐European individuals and to patients with SLD and advanced liver fibrosis, when the amount of steatosis tends to decrease in parallel with disease progression. Looking ahead, the quality of MRI images strongly influences the prediction outcomes, especially when using images directly for prediction. For future research, we recommend utilising higher‐resolution MRI 2D images or even 3D liver data, as these provide more detailed information. Models, trained on these more comprehensive datasets, may achieve higher prediction accuracy by learning from the additional information.

Further, we assessed model performance using a five‐fold cross‐validation approach, ensuring that 15% of the data was randomly selected in a stratified manner and set aside as an internal independent test set, maintaining the same distribution as the training set. Due to the unavailability of an external cohort for validation, this internal test set was used to assess the model's generalizability. We recognise this as a limitation of our study and recommend that future work include external validation in an independent cohort to further confirm these findings. Furthermore, integrating radiomics data, extracted from liver regions, alongside imaging data, could further enhance prediction performance. The integration of radiomics and imaging data would yield a comprehensive set of features, leading to more robust and accurate predictive models.

## 5 Conclusion

Our research represents a novel advancement in the non‐invasive identification of *PNPLA3* I148M associated with SLD using deep learning models applied to MRI images. The ability to non‐invasively predict the presence of the *PNPLA3* I148M variant using MRI images can enhance early detection and risk stratification. The accuracy of this approach is expected to improve further when combined with tabular data, like diagnoses or serum values. With further independent validation, this method has the potential for immediate integration into clinical practice, offering real‐time selection of a high‐risk population for genetic risk assessment during routine imaging.

## Author Contributions

Y.C. developed the code, performed data analysis, prepared all figures and tables, and wrote the main manuscript text. D.T. and C.V.S. designed the study and experiments, providing overall supervision. D.H. provided the water‐fat separation toolbox and the related processing code. B.P.M.L. offered critical guidance and contributed to revisions throughout the writing process. T.L. provided support for MRI image analysis and contributed to manuscript revisions. G.A.M.‐F. and T.S. offered technical advice on code development. C.D. assisted in the validation of liver segmentation. All authors critically reviewed the manuscript content and approved the final version.

## Conflicts of Interest

R.L. serves as a consultant to Aardvark Therapeutics, Altimmune, Arrowhead Pharmaceuticals, AstraZeneca, Cascade Pharmaceuticals, Eli Lilly, Gilead, Glympse bio, Inipharma, Intercept, Inventiva, Ionis, Janssen Inc., Lipidio, Madrigal, Neurobo, Novo Nordisk, Merck, Pfizer, Sagimet, 89 bio, Takeda, Terns Pharmaceuticals and Viking Therapeutics. R.L. has stock options in Sagimet biosciences. In addition, his institution received research grants from Arrowhead Pharmaceuticals, Astrazeneca, Boehringer‐Ingelheim, Bristol‐Myers Squibb, Eli Lilly, Galectin Therapeutics, Gilead, Intercept, Hanmi, Intercept, Inventiva, Ionis, Janssen, Madrigal Pharmaceuticals, Merck, Novo Nordisk, Pfizer, Sonic Incytes and Terns Pharmaceuticals. Co‐founder of LipoNexus Inc. J.N.K. declares consulting services for Bioptimus, France; Panakeia, UK; AstraZeneca, UK; and MultiplexDx, Slovakia. Furthermore, he holds shares in StratifAI, Germany, Synagen, Germany, and Ignition Lab, Germany; has received an institutional research grant by GSK; and has received honoraria by AstraZeneca, Bayer, Daiichi Sankyo, Eisai, Janssen, Merck, MSD, BMS, Roche, Pfizer, and Fresenius. L.V. declares speaking for: Viatris, Novo Nordisk, GSK, consulting for: Novo Nordisk, Pfizer, Boehringer Ingelheim, Resalis, MSD. All other authors declare no financial or non‐financial competing interests.

## Supporting information


Data S1.

## Data Availability

The data that support the findings of this study are available from UK Biobank. Restrictions apply to the availability of these data, which were used under license for this study. Data are available from https://www.ukbiobank.ac.uk/ with the permission of UK Biobank.
