# Supplementary material for: Deep Learning Reveals Liver MRI Features Associated With PNPLA3 I148M in Steatotic Liver Disease
Source: Liver Int. 2025 Jun 6;45(7):e70164. doi: 10.1111/liv.70164 (PMC12143367; doi:10.1111/liv.70164)
Supplement: Supplementary file 1 — Data S1. [file LIV-45-0-s001.docx]

**Supplementary**

**Supplementary Table 1: Label counts for different test sets**

| Label counts/  Result | Training Set  (PNPLA3), n | Test Set | | | | |
| --- | --- | --- | --- | --- | --- | --- |
|  |  | PNPLA3, n | Obesity, n | Type 2 Diabetes, n | C_1, n | C_2, n |
| Total | 6751 | 1192 | 1192 | 1190 | 1192 | 1192 |
| Positive | 817 | 144 | 428 | 144 | 490 | 83 |
| Negative | 5934 | 1048 | 764 | 1046 | 702 | 1109 |
| AUROC |  | 0.68 | 0.35 | 0.47 | 0.37 | 0.39 |

In the test set, "positive" and "negative" indicate the numbers of homozygous carriers and non-carriers when PNPLA3 I148M is the outcome, obese (BMI ≥ 30) and non-obese (BMI < 30) when obesity is the outcome, and individuals with and without type 2 diabetes when type 2 diabetes is the outcome. C_1 and C_2 are composite labels considering that the potential combined influence of these risk factors, C_1 was defined as positive if either the diabetes label or the obesity label was positive, while C_2 was defined as positive only when both obesity and diabetes were positive.

**Supplementary Table 2: AUROC in the SLD group without excessive alcohol consumption**

| **Cohort** | Number of samples | | AUROC in test set (95% CI) |
| --- | --- | --- | --- |
|  | Homozygote | Non-carrier |  |
| SLD group | 961 | 6982 | 0.68 (0.64-0.73) |
| SLD group without excessive alcohol consumption | 900 | 6491 | 0.67 (0.63-0.71) |

**Supplementary Figure 1: Benchmarking of different machine learning models using AUROCs**


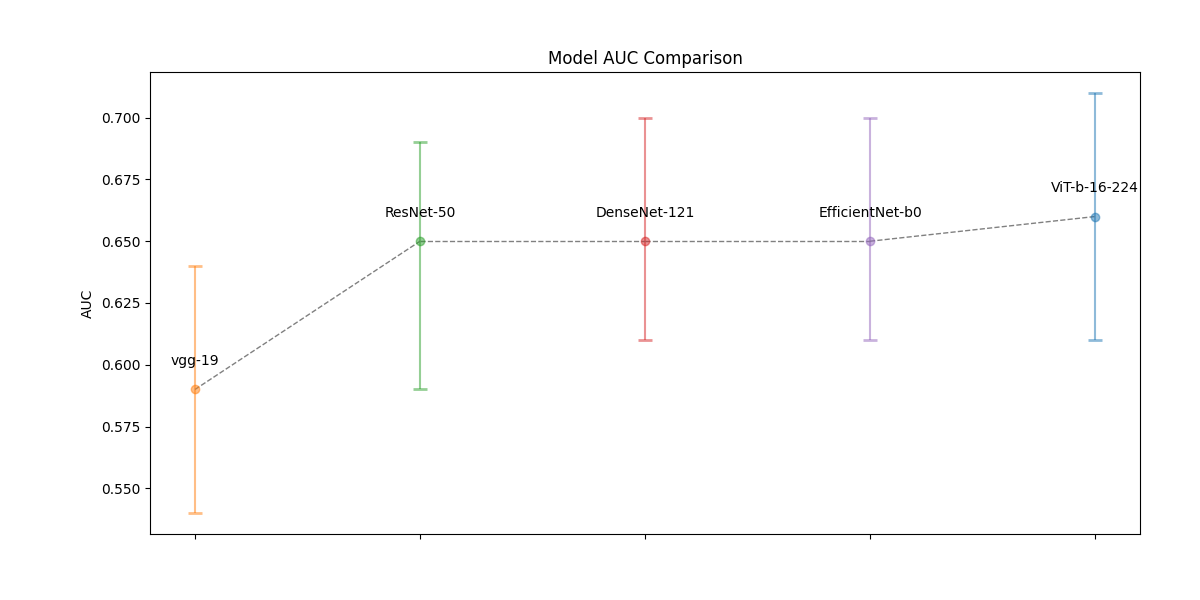


We evaluated the performance of five different models based on patients in the SLD group with the *PNPLA3* I148M variant: ViT-b-16-224, VGG-19, ResNet-50, DenseNet-121, and EfficientNet-b0. Among these, the ViT-b-14-224 model demonstrated the highest AUROC of 0.66 (95% CI: 0.61-0.71), indicating the strongest discriminative ability. The other models yielded AUROC values of the were 0.59 (95% CI: 0.54-0.64) for vgg-19, 0.65 (95% CI: 0.59-0.69) for ResNet-50, 0.65 (95% CI: 0.61-0.70) for DenseNet-121, and 0.65 (95% CI: 0.61-0.70) for EfficientNet-b0. Although the AUROCs of these models were relatively close, we ultimately selected the ViT-b-16-224 model as the optimal choice due to its superior performance and its Transformer-based architecture providing greater scalability and adaptability. Additionally, the architectural features make the ViT model advantageous for future model optimization and application expansion.

**Supplementary Figure 2: Comparison of transformer-based prediction of hepatic steatosis-related SNPs on liver MRIs in patients with and without steatotic liver disease**


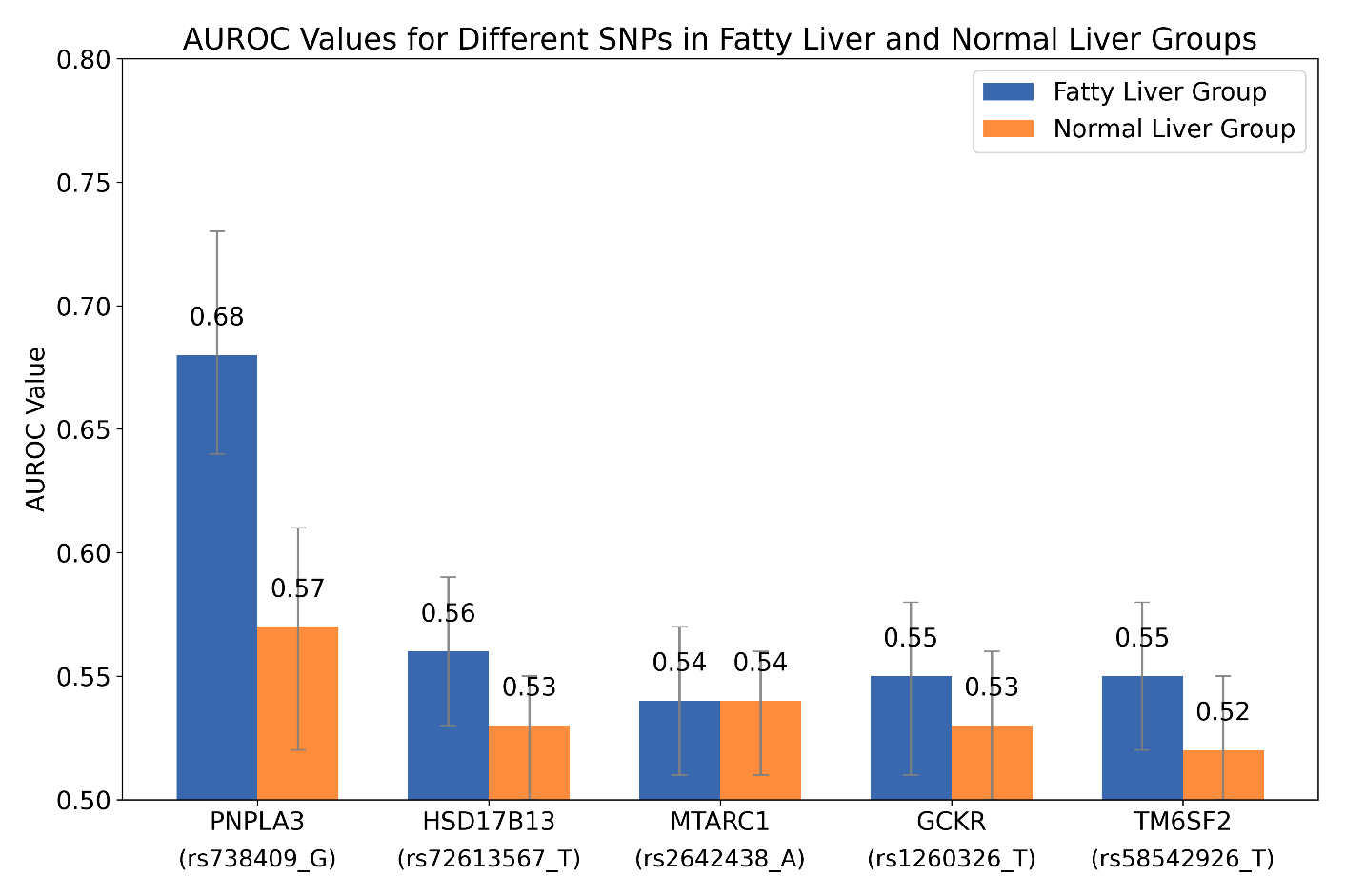
To investigate the predictive performance of the ViT-b-16-224 model across different SNPs and populations, we finetuned the pretrained model on SNPs associated with SLD disease in both SLD and non-SLD groups. The figure displays the AUROCs and their 95% confidence intervals (CI) for each SNP in both groups. In the SLD group, the AUROCs for *PNPLA3* I148M, *HSD17B13 (rs72613567_T)*, *MTARC1 (rs2642438_A)*, *GCKR (rs1260326_T)*, and *TM6SF2 (rs58542926_T)* were 0.68 (95% CI: 0.64-0.73), 0.56 (95% CI: 0.53-0.59), 0.54 (95% CI: 0.51-0.57), 0.55 (95% CI: 0.51-0.58), and 0.55 (95% CI: 0.52-0.58), respectively. In the non-SLD group, the corresponding AUROCs were 0.57 (95% CI: 0.52-0.61), 0.53 (95% CI: 0.50-0.55), 0.54 (95% CI: 0.51-0.56), 0.53 (95% CI: 0.50-0.56), and 0.52 (95% CI: 0.50-0.55). The AUROCs in the SLD group are generally higher than those in the non-SLD group, with *PNPLA3* I148M demonstrating better predictive value, achieving an AUROC of 0.68 compared to 0.57 in the non-SLD group. This indicates that *PNPLA3* I148M has a higher predictive value for SLD disease in the SLD population.
